# Causal relationship between trunk and lower limb fat mass and intervertebral disc disorders: A 2-sample Mendelian randomization study

**DOI:** 10.1097/MD.0000000000042884

**Published:** 2025-07-18

**Authors:** Zhihao Huang, Zhiqi Tian, Kunzong Tian, Yongming Wang, Yuting Jiang

**Affiliations:** a School of Big Data and Fundamental Sciences, Shandong Institute of Petroleum and Chemical Technology, Dongying, China; b Department of Clinical Laboratory, Shengli Oilfield Central Hospital, Dongying, China.

**Keywords:** causal relationship, fat mass, intervertebral disc disorders, Mendelian randomization, single nucleotide polymorphism

## Abstract

Intervertebral disc disorders (IVDDs) are a major cause of disability worldwide, influenced by genetic and lifestyle factors such as obesity. Although the role of body fat distribution in IVDDs is recognized, the causal relationship remains unclear. This study aimed to elucidate the genetic basis of this relationship by examining the association between fat mass distribution in the trunk and lower limbs and the risk of IVDDs using Mendelian randomization (MR). The study utilized single nucleotide polymorphism (SNP) as instrumental variable to investigate genetic predispositions to increased fat mass in specific body regions and their associations with IVDDs. Initially, 98,51,866 SNPs for trunk and lower limb fat mass and 21,304,570 SNPs for IVDDs were analyzed. Data cleaning steps, including linkage disequilibrium clumping, SNP merging, allele harmonization, and checks against the genome-wide association studies catalog database, reduced the number of relevant SNPs to 353 for trunk fat mass, and 346 and 337 for left and right leg fat mass, respectively. The causal analysis was conducted using the inverse variance weighted (IVW) method and MR-Egger method, with sensitivity analyses to test robustness. The IVW method showed a significant positive causal relationship between trunk fat mass and IVDDs (odds ratio (OR) = 1.274, 95% confidence interval (CI): 1.186–1.368, *P* < .001), whereas the MR-Egger method did not show statistical significance (*P* = .207). Both methods revealed a consistent and significant association for lower limb fat mass with IVDDs. Specifically, the IVW method indicated odds ratios of 1.454 (95% CI: 1.323–1.597, *P* < .001) for left leg fat mass and 1.467 (95% CI: 1.332–1.616, *P* < .001) for right leg fat mass. The presence of heterogeneity and potential pleiotropy was assessed, supporting the stability and reliability of the causal inferences. This study confirmed a positive causal relationship between trunk and lower limb fat mass and the risk of IVDDs, emphasizing the need to consider body fat distribution in IVDD prevention and management. The findings suggested that reducing trunk and lower limb fat mass could lower IVDD risk. This research provided valuable insights into the genetic and physiological links between body fat distribution and IVDDs, paving the way for targeted preventive strategies and therapeutic interventions.

## 1. Introduction

In recent years, the incidence of low back pain and neck pain has been increasing annually, becoming more widespread and affecting younger populations, significantly disrupting people’s work and daily lives.^[1,2]^ In Europe, low back pain is the leading cause of medically certified sick leave and early retirement. However, the burden of work disability due to low back pain varies significantly across European countries. For instance, in Norway and Sweden in the year 2000, the short-term sickness absence rates among individuals with back pain were comparable (5.1% and 6.4%, respectively); however, the prevalence of long-term medically certified sickness absence differed markedly, with rates of 22% in Norway and 15% in Sweden. In the United States, low back pain is the primary contributor to lost workdays among all occupational musculoskeletal disorders. In 1999, 58 out of every 10,000 US workers filed a back pain-related compensation claim, whereas the corresponding figure in Japan during the same period was markedly lower, at only 1 per 10,000 workers.^[3]^ These conditions are now recognized as the foremost causes of disability worldwide,^[4,5]^ with intervertebral disc disorders (IVDDs) identified as the primary culprits.^[6,7]^ Given the widespread nature and the significant strain these IVDDs-related diseases place on healthcare systems, there is a pressing and critical need to deepen our understanding of IVDDs.^[8]^ The intervertebral disc serves as a moderately moving joint nestled between the vertebrae, playing a crucial role in imparting flexibility and facilitating load distribution throughout the spinal column. This disc is intricately composed of various interdependent tissues: the nucleus pulposus at its center, characterized by high hydration levels; the encircling annulus fibrosus, notable for its elasticity and fibrous nature; and the cartilaginous endplate, which establishes a connection to the vertebral bodies.^[9]^ Each tissue type not only performs a unique function but also boasts a distinct matrix structure, sustained by a specialized cell population exhibiting unique phenotypic characteristics. In a healthy state, the intervertebral disc adeptly manages the delicate equilibrium of matrix turnover, balancing synthesis and degradation. However, this equilibrium is frequently disrupted, precipitating degenerative conditions. IVDDs represent a multifaceted ailment, where environmental influences and a plethora of genes likely converge, crafting a comprehensive degenerative phenotype.^[10]^ Obesity is identified as one of the primary contributing factors to the degeneration of intervertebral discs,^[11]^ with research into its mechanisms focusing on aspects such as fat infiltration in the trunk region, the effects of excessive stress, and the impact of adipokines released. The upright posture of humans subjects the intervertebral discs to various loads including tensile forces, compression, and fluid shear stress. Factors like excessive obesity lead to increased body mass and chronic wear, inevitably resulting in damage to the discs and disruption to adjacent structures. This brings about a biomechanical imbalance, ultimately manifesting in clinical symptoms. The release of adipocyte factors, notably leptin and adiponectin, plays a critical role in driving the metabolic changes and inflammatory responses associated with disc degeneration.^[12,13]^ Furthermore, obesity is linked to significantly elevated levels of IL-6 and pro-inflammatory cascades throughout the body, contributing to the inflammation-mediated pathway of disc degeneration.^[14]^

Fat mass is widely utilized to evaluate the risk of developing various diseases. However, despite being a strong correlate of body mass index (BMI), its use in the context of IVDDs has been less frequently reported. Studies have validated that the subcutaneous fat tissue thickness at the L1-L2 level as superior to BMI in predicting spine degeneration.^[15]^ Research utilizing lumbar spine magnetic resonance imaging to compare BMI, abdominal diameter, sagittal abdominal diameter, and subcutaneous abdominal fat thickness with Pfirrmann grading of disc degeneration has revealed that, compared to BMI, the amount of abdominal fat was a more dependable metric for assessing the extent of disc degeneration in patients.^[16]^ This indicated that beyond the sheer volume of fat, its distribution across the body had varying impacts, though the precise causal relationships remained to be further explored.

In the investigation of pathogenic factors and disease risk, randomized controlled trial (RCT) are highly regarded for their reference value, yet their widespread application is hampered by various clinical constraints. Mendelian randomization (MR), however, represents a methodology that utilizes genetic variants as instrumental variables.^[17]^ Following Mendelian laws – that alleles are randomly assorted and fixed at the time of gamete formation – it circumvents confounding factors, offering a means to explore the causal relationship between exposure and outcome.^[18,19]^ This approach can be likened to RCTs, with the key distinction being that while randomization in RCT occurs at the trial’s initiation, in MR, it happens during gamete formation and conception.^[20]^ This study was devised that selectively focused on the fat mass in the trunk and both lower limbs, excluding total body and upper limb fat mass. This approach aimed to utilize MR to examine the causal relationship between the distribution of fat and IVDDs. This strategic selection underscored the objective to clarify the causative links between fat distribution and IVDDs, leveraging the unique insights afforded by MR.

## 2. Materials and methods

### 2.1. Study design

**Figure 1.**
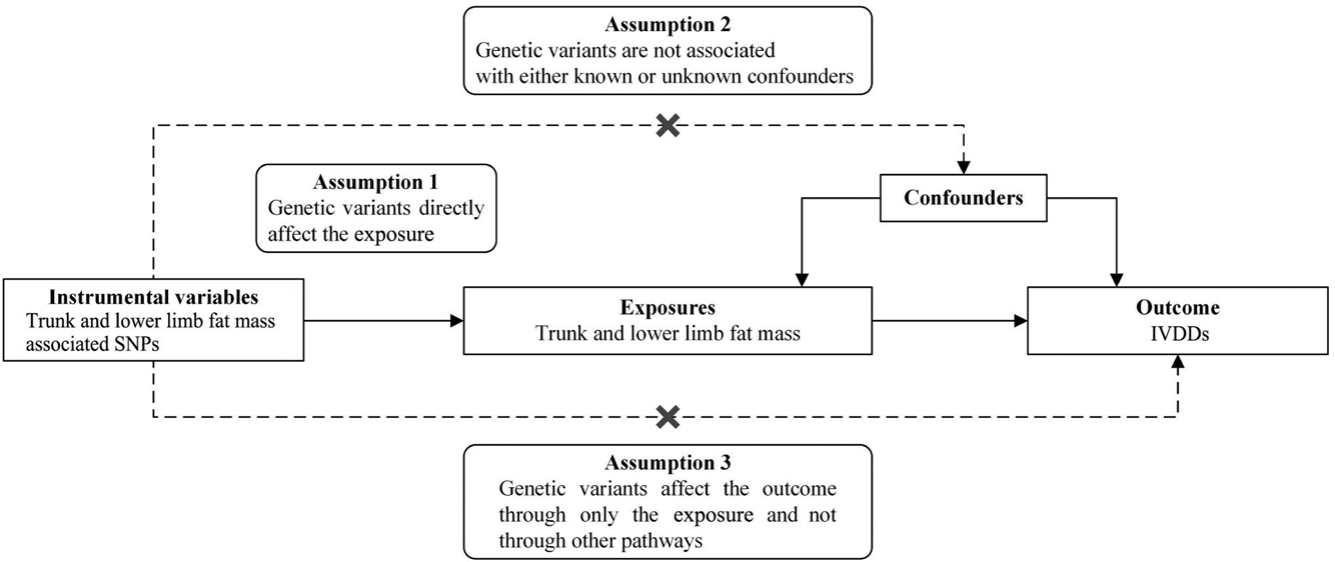
Schematic diagram of the MR assumptions. IVDDs = intervertebral disc disorders, MR = Mendelian randomization, SNP = single nucleotide polymorphism.

**Figure 2.**
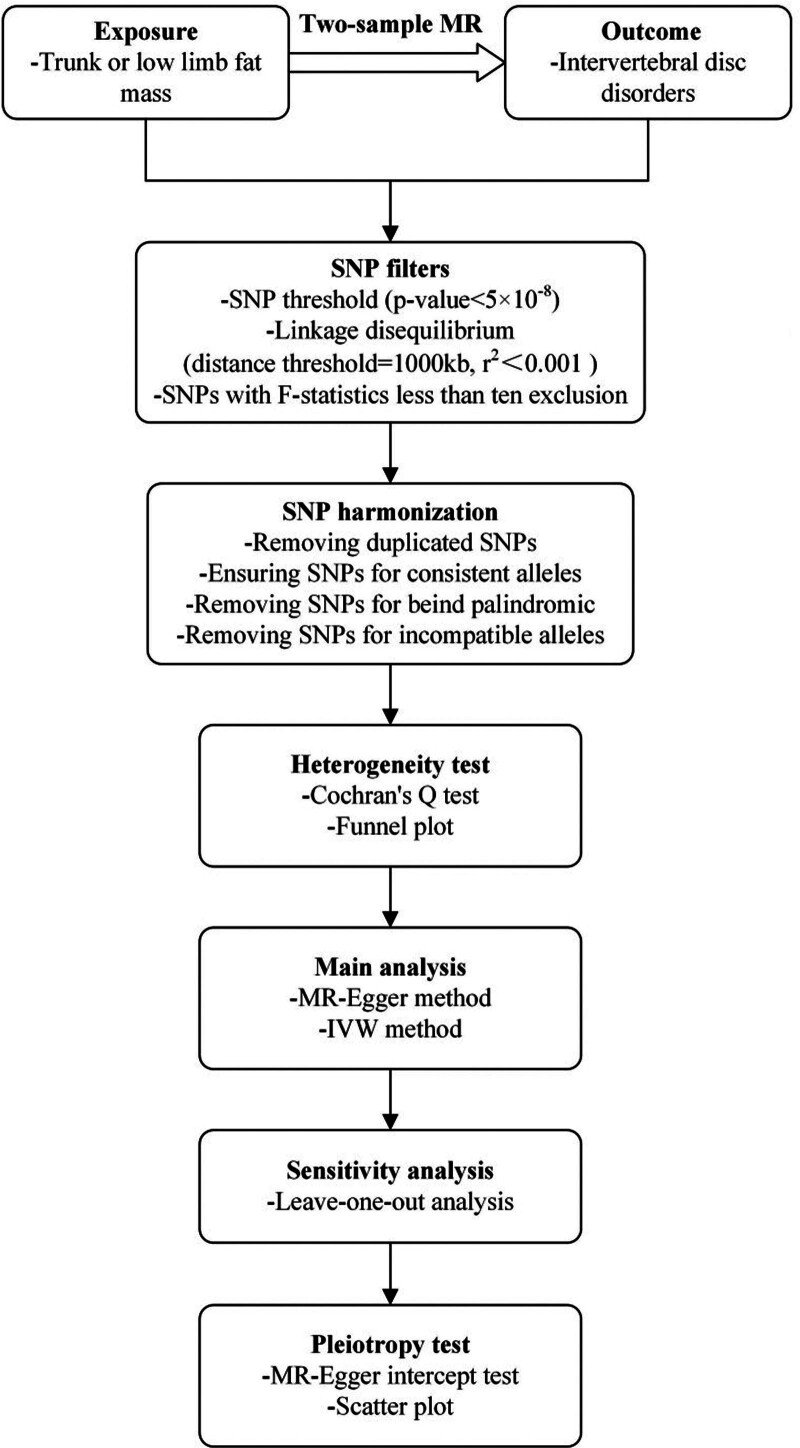
The steps of MR analysis. IVW = inverse variance weighted, MR = Mendelian randomization, SNP = single nucleotide polymorphism.

This study was conducted in accordance with Strengthening the Reporting of Observational Studies in Epidemiology Using MR Statement.^[21]^ The study identified IVDDs as the outcome, selecting single nucleotide polymorphism (SNP) significantly associated with trunk and lower limb fat mass as instrumental variables. These were utilized to explore the causal relationship between the exposure and the outcome using MR analysis. To ensure the validity of the MR analysis, 3 critical assumptions were established^[22]^: genetic variants directly affect the exposure; genetic variants are not associated with either known or unknown confounders; genetic variants affect the outcome through only the exposure and not through other pathways (Fig. 1). To corroborate the robustness of the findings, the study employed heterogeneity test, sensitivity analysis, and pleiotropy test, thus enhancing the reliability of the results^[23]^ (Fig. 2).

### 2.2. Data sources

The primary sources included the Medical Research Council (MRC), Integrative Epidemiology Unit (IEU), open genome-wide association studies (GWAS; https://gwas.mrcieu.ac.uk), and the FinnGen Biobank (https://storage.googleapis.com/finngen-public-data-r10/summary_stats/finngen_R10_M13_INTERVERTEB.gz). Genetic data for trunk and bilateral lower limb fat mass were obtained from the MRC IEU GWAS database. All genetic data were from the MRC-IEU Consortium, with data for trunk fat mass (GWAS ID: ukb-b-20044), left leg fat mass (GWAS ID: ukb-b-7212), and right leg fat mass (GWAS ID: ukb-b-18096) all stemming from research conducted in 2018. The trunk fat mass dataset included 4,54,588 samples and 98,51,867 SNPs, the left leg fat mass dataset included 4,54,823 samples and 98,51,867 SNPs; the right leg fat mass dataset included 4,54,846 samples and 98,51,867 SNPs. Genetic data for IVDDs were obtained from the FinnGen Biobank database. And genetic data were from The latest 10th edition of the FinnGen Biobank Consortium, with data for IVDDs (GWAS ID: finngen_R10_M13_INTERVERTEB) stemming from research conducted in 2023. The IVDDs dataset comprised 3,36,439 samples and 21,304,570 SNPs. All studies’ sample populations originated from Europe (Table 1).

**Table 1:** Summary information of GWAS data in MR studies.

Trait	Uniform Resource Locator of the data source	GWAS ID	Ethnic origin of the sample	Sample size	Number of SNPs	Consortium	Year
Trunk fat mass	https://gwas.mrcieu.ac.uk	ukb-b-20044	Europe	4,54,588	9,851,867	MRC-IEU	2018
Left leg fat mass	https://gwas.mrcieu.ac.uk	ukb-b-7212	Europe	4,54,823	9,851,867	MRC-IEU	2018
Right leg fat mass	https://gwas.mrcieu.ac.uk	ukb-b-18096	Europe	4,54,846	9,851,867	MRC-IEU	2018
IVDDs	https://storage.googleapis.com/finngen-public-data-r10/summary_stats/finngen_R10_M13_INTERVERTEB.gz	finngen_R10_M13_INTERVERTEB	Europe	3,36,439	21,304,570	the latest 10th edition of the FinnGen Biobank	2023

GWAS = genome-wide association studies, IEU = Integrative Epidemiology Unit, IVDDs = intervertebral disc disorders, MR = Mendelian randomization, MRC = Medical Research Council, SNP = single nucleotide polymorphism.

### 2.3. Selection of genetic variants

As mentioned above, genetic variants employed in MR analysis must fulfill 3 fundamental assumptions. To adhere to the first assumption, genome-wide significant SNPs (*P* < 5 × 10^−8^) were selected as genetic instruments for MR analysis,^[24]^ with variants being clumped based on an *r*^2^ measure of linkage disequilibrium <0.001 within a 10,000 kb window,^[25]^ ensuring a robust selection process. For the second assumption, the study meticulously excluded SNPs associated with potential confounders by manually reviewing the GWAS Catalog database (https://www.ebi.ac.uk/gwas/), ensuring the purity of the instrumental variables.^[26]^ In fulfilling the third assumption, SNPs strongly associated with IVDDs (*P* < 5 × 10^−8^) were also excluded, further refining the instrument’s validity. Additionally, the strength of the selected instrumental variables was assessed by calculating the *F*-statistic to rule out weak instrument bias, with an *F* > 10 indicating the absence of such bias, thereby reinforcing the causal inference.^[27]^ The *F*-statistic is calculated using the formula: *F* = [*R*^2^(N − 2)]/(1 − *R*^2^), where *R*^2^ represents the proportion of the variability in trunk and lower limb fat mass explained by each instrument, and N is the sample size of the GWAS for the SNP-trunk and lower limb fat mass association. The calculation of *R*^2^ is as follows: *R*^2^ = [2 × EAF × (1 − EAF) × β^2^]/[2 × EAF × (1 − EAF) × β^2^ + 2 × EAF × (1 − EAF) × N × SE(β)^2^]. Here, EAF is the effect allele frequency, β is the estimated genetic effect on trunk and lower limb fat mass, and SE(β) is the standard error of the genetic effect.^[28]^

### 2.4. Statistical analysis

Using the TwoSample MR package in R software version 4.4.2 for data analysis, through the inverse variance weighted (IVW) method and the MR-Egger method, the odds ratio (OR) and confidence interval (CI) in the regression models are used to assess the causal relationship between trunk and lower limb fat mass and IVDDs. The statistical analysis to estimate causal relationships were regarded as statistically significant at a Bonferroni corrected *P* < .017 (0.05/3; *P* = .05 adjusted for 3 tests).^[29]^ The *P*-value was set at .05 for statistical significance for both heterogeneity and pleiotropy analysis.

## 3. Result

### 3.1. Causal relationship between trunk fat mass and intervertebral disc disorders

**Figure 3.**
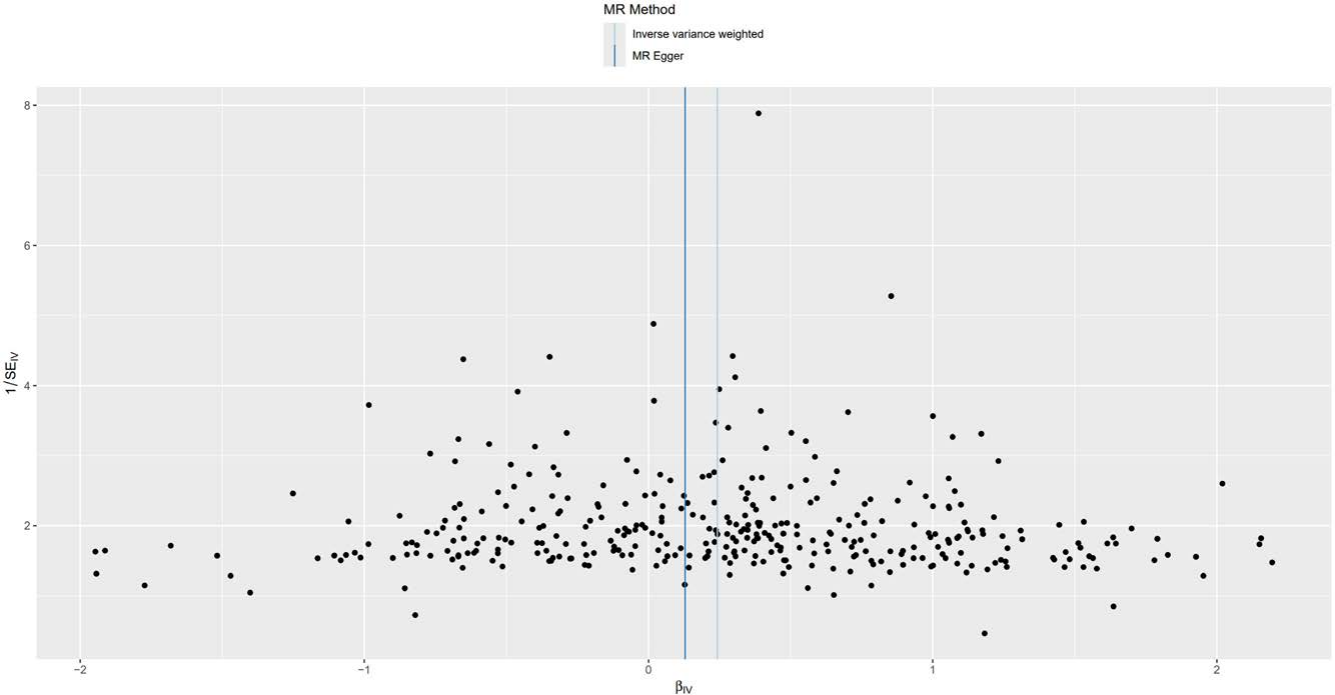
Funnel plot for MR analysis of trunk fat mass and IVDDs. IVDDs = intervertebral disc disorders, MR = Mendelian randomization.

**Figure 4.**
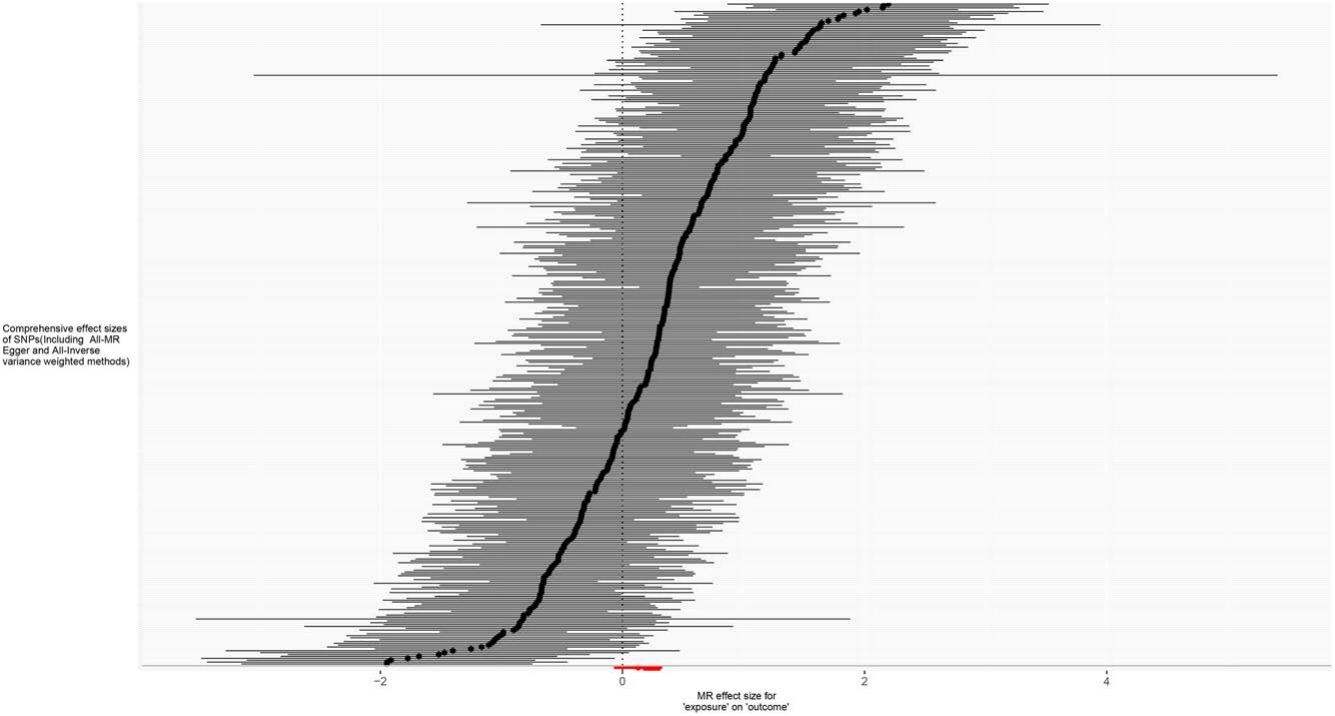
Forest plot for MR analysis of trunk fat mass and IVDDs. IVDDs = intervertebral disc disorders, MR = Mendelian randomization, SNP = single nucleotide polymorphism.

**Figure 5.**
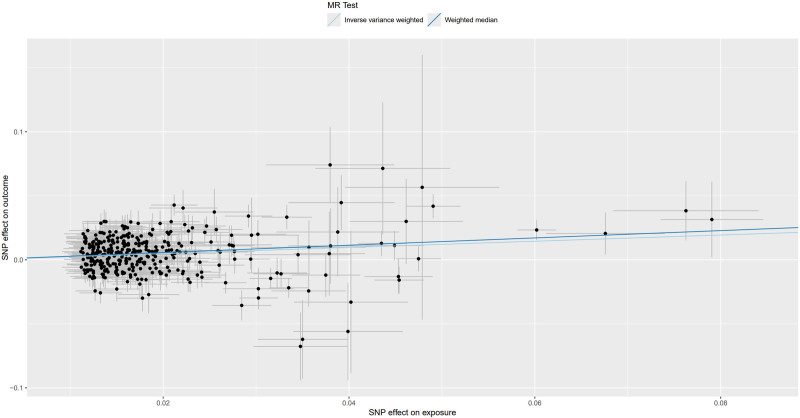
Scatter plot for MR analysis of trunk fat mass and IVDDs. IVDDs = intervertebral disc disorders, MR = Mendelian randomization, SNP = single nucleotide polymorphism.

**Figure 6.**
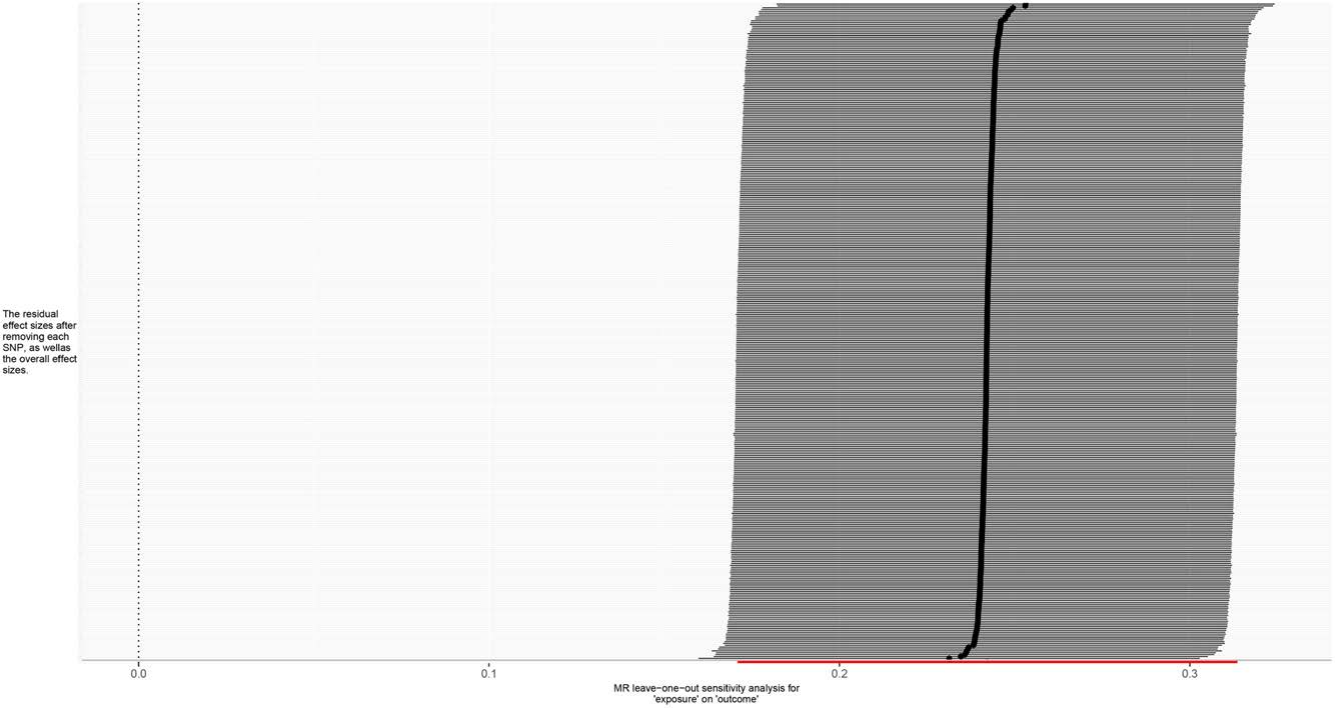
Leave-one-out forest plot for MR analysis of trunk fat mass and IVDDs. IVDDs = intervertebral disc disorders, MR = Mendelian randomization, SNP = single nucleotide polymorphism.

For trunk fat mass, 98,51,866 SNPs were read, and after using the clumping function to remove linkage disequilibrium, 423 SNPs strongly associated with trunk fat mass remained (*P* < 5 × 10^−8^). For IVDDs, 21,304,570 SNPs were read, and after merging with the SNPs strongly associated with trunk fat mass using the merge function, 414 SNPs remained. After removing 2 SNPs strongly associated with IVDDs, 412 SNPs were left. Using the harmonize function to eliminate duplicated, palindromic, and incompatible alleles SNPs, 353 SNPs remained. A review of the GWAS Catalog database found no interference from confounding factors, and the calculation of the *F*-statistic did not reveal any weak instrumental variables. Therefore, 353 SNPs were ultimately included as genetic variants for assessing the causal relationship between trunk fat mass and IVDDs. The Cochran’s *Q* test for the IVW method showed significant heterogeneity (*P* < .001), as did the Cochran’s *Q* test for the MR-Egger method (*P* < .001). Combined with the asymmetry observed in the funnel plot (Fig. 3), these results indicated the presence of heterogeneity in the study, necessitating the use of a random-effect model for statistical analysis. The research result from the IVW method was as follows: OR = 1.274, 95% CI: 1.186–1.368, *P* < .001, indicating statistical significance; whereas the result from the MR-Egger method was: OR = 1.138, 95% CI: 0.932–1.389, *P* = .206, showing no statistical significance (Fig. 4 and Table S1, Supplemental Digital Content, https://links.lww.com/MD/P370). Despite the MR-Egger method’s result not reaching statistical significance, the direction of effect was consistent across both methods. With the IVW method’s result taking precedence, it could be concluded that an increase in trunk fat mass was a risk factor for the occurrence of IVDDs, indicating a positive causal relationship between trunk fat mass and IVDDs. The MR-Egger intercept test result (*P* = .235), along with the scatter plot (Fig. 5) showing a *y*-axis intercept close to 0, suggested the absence of potential pleiotropy in the study. A “leave-one-out” sensitivity analysis was conducted to examine whether the exclusion of individual SNPs would affect the overall causal inference. The analysis revealed that no individual SNP significantly impacted the prediction of the causal relationship (Fig. 6 and Table S2, Supplemental Digital Content, https://links.lww.com/MD/P370), indicating that the study’s result was relatively stable.

### 3.2. Causal relationship between left leg fat mass and intervertebral disc disorders

**Figure 7.**
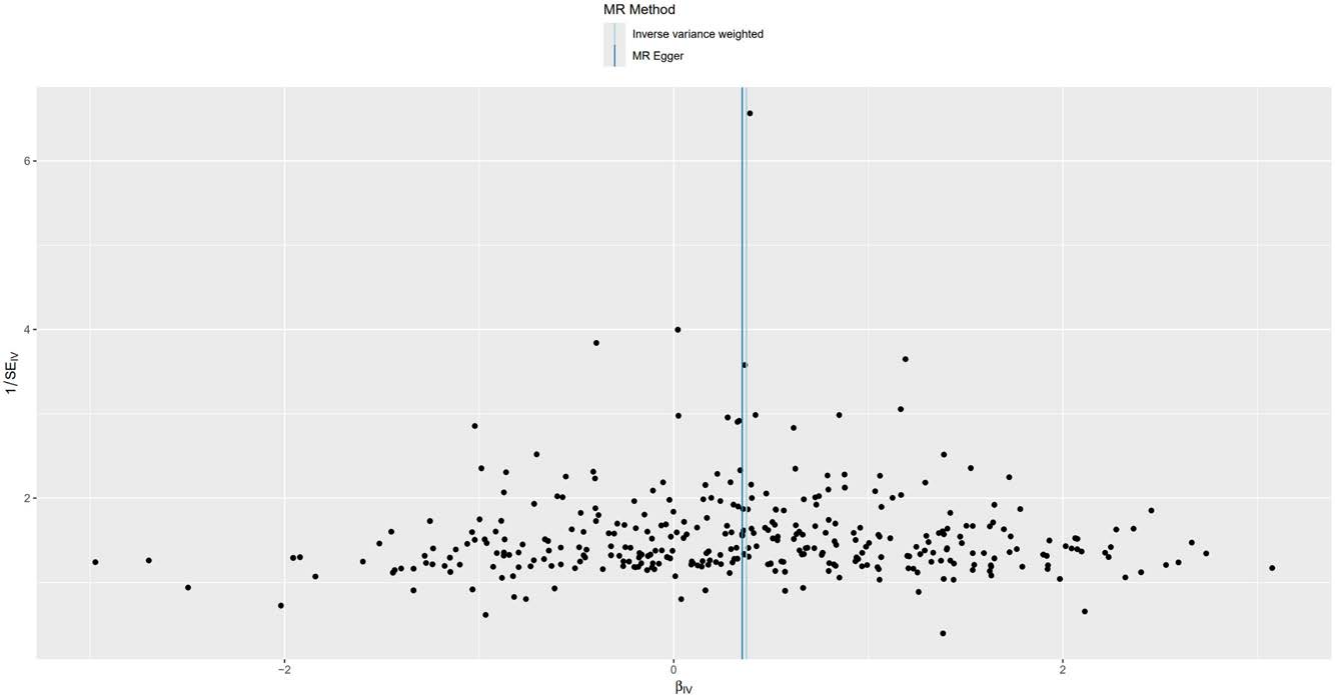
Funnel plot for MR analysis of left leg fat mass and IVDDs. IVDDs = intervertebral disc disorders, MR = Mendelian randomization.

**Figure 8.**
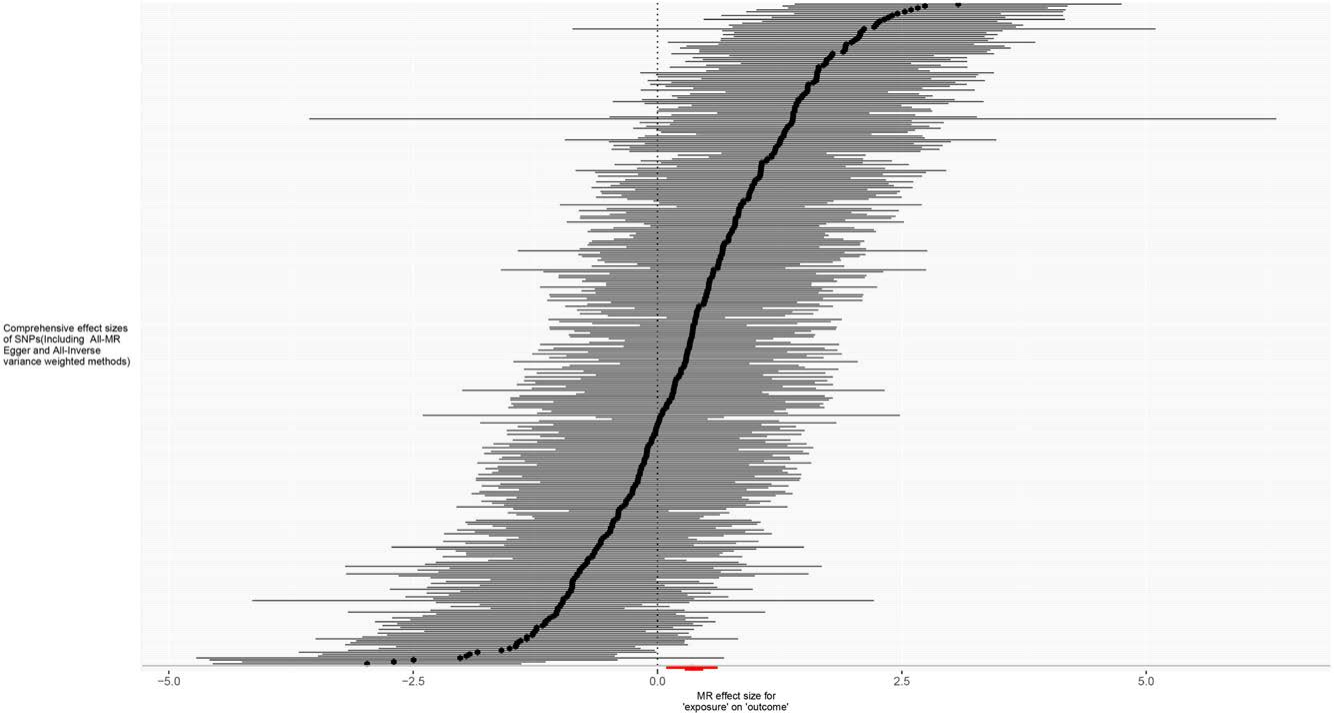
Forest plot for MR analysis of left leg fat mass and IVDDs. IVDDs = intervertebral disc disorders, MR = Mendelian randomization, SNP = single nucleotide polymorphism.

**Figure 9.**
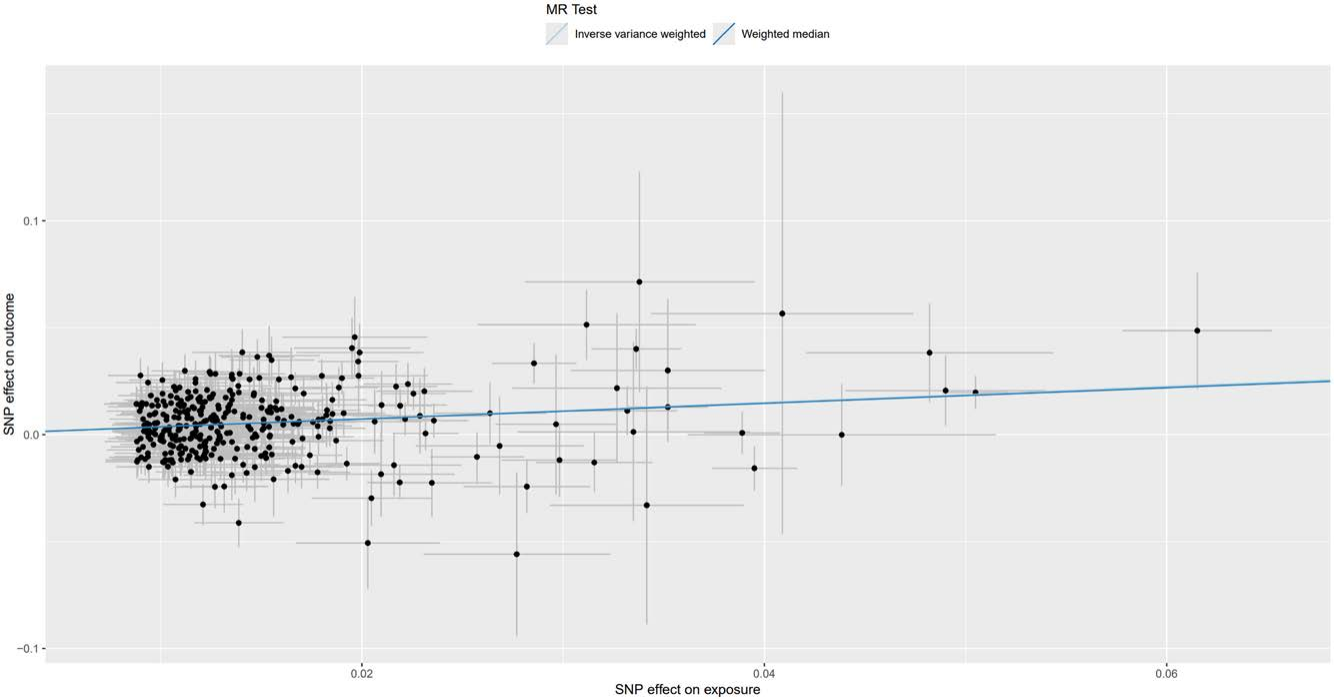
Scatter plot for MR analysis of left leg fat mass and IVDDs. IVDDs = intervertebral disc disorders, MR = Mendelian randomization, SNP = single nucleotide polymorphism.

**Figure 10.**
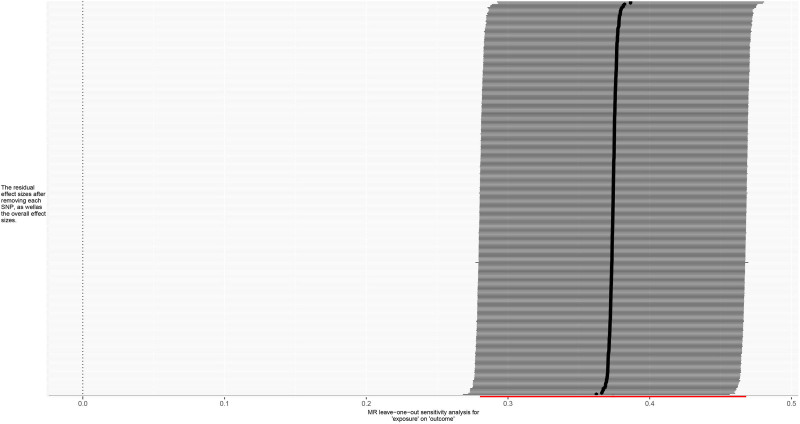
Leave-one-out forest plot for MR analysis of left leg fat mass and IVDDs. IVDDs = intervertebral disc disorders, MR = Mendelian randomization, SNP = single nucleotide polymorphism.

For left leg fat mass, 98,51,866 SNPs were read, and after using the clumping function to remove linkage disequilibrium, 421 SNPs strongly associated with left leg fat mass remained (*P* < 5 × 10^−8^). For IVDDs, 21,304,570 SNPs were read, and after merging with the SNPs strongly associated with left leg fat mass using the merge function, 412 SNPs remained. Using the harmonize function to eliminate duplicated, palindromic, and incompatible alleles SNPs, 346 SNPs remained. A review of the GWAS Catalog database found no interference from confounding factors, and the calculation of the *F*-statistic did not reveal any weak instrumental variables. Therefore, 346 SNPs were ultimately included as genetic variants for assessing the causal relationship between left leg fat mass and IVDDs. The Cochran’s *Q* test for the IVW method showed significant heterogeneity (*P* < .001), as did the Cochran’s *Q* test for the MR-Egger method (*P* < .001). Combined with the asymmetry observed in the funnel plot (Fig. 7), these results indicated the presence of heterogeneity in the study, necessitating the use of a random-effect model for statistical analysis. The research result from the IVW method was as follows: OR = 1.454, 95% CI: 1.323–1.597, *P* < .001, indicating statistical significance. Similarly, the result from the MR-Egger method was: OR = 1.423, 95% CI: 1.094–1.851, *P* = .009, showing statistical significance (Fig. 8 and Table S3, Supplemental Digital Content, https://links.lww.com/MD/P370). The direction of effect was consistent across both methods. With the IVW method’s result taking precedence, it could be concluded that an increase in left leg fat mass was a risk factor for the occurrence of IVDDs, indicating a positive causal relationship between left fat mass and IVDDs. The MR-Egger intercept test result (*P* = .864), along with the scatter plot (Fig. 9) showing a *y*-axis intercept close to 0, suggested the absence of potential pleiotropy in the study. A “leave-one-out” sensitivity analysis was conducted to examine whether the exclusion of individual SNPs would affect the overall causal inference. The analysis revealed that no individual SNP significantly impacted the prediction of the causal relationship (Fig. 10 and Table S4, Supplemental Digital Content, https://links.lww.com/MD/P370), indicating that the study’s result was relatively stable.

### 3.3. Causal relationship between right leg fat mass and intervertebral disc disorders

**Figure 11.**
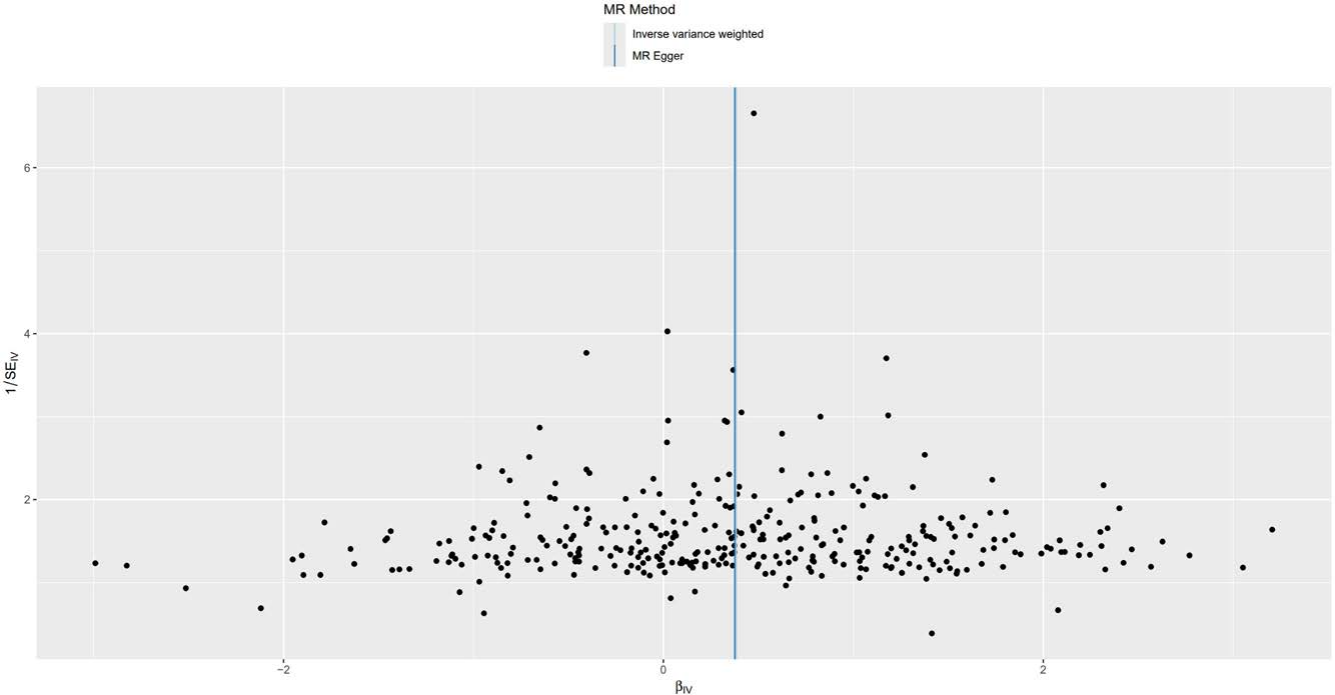
Funnel plot for MR analysis of right leg fat mass and IVDDs. IVDDs = intervertebral disc disorders, MR = Mendelian randomization.

**Figure 12.**
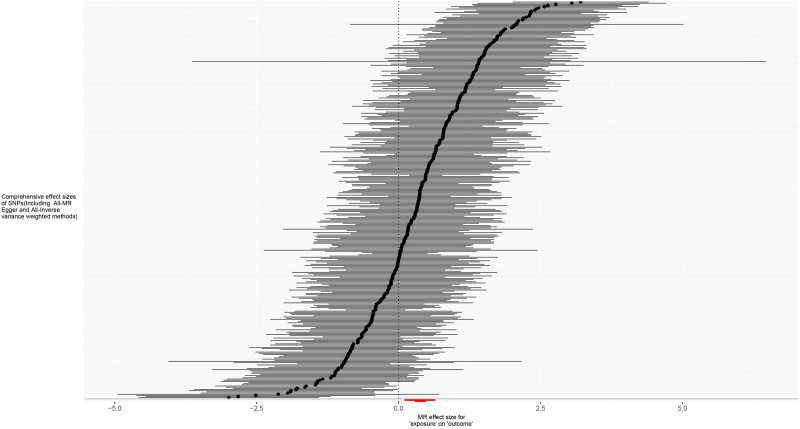
Forest plot for MR analysis of right leg fat mass and IVDDs. IVDDs = intervertebral disc disorders, MR = Mendelian randomization, SNP = single nucleotide polymorphism.

**Figure 13.**
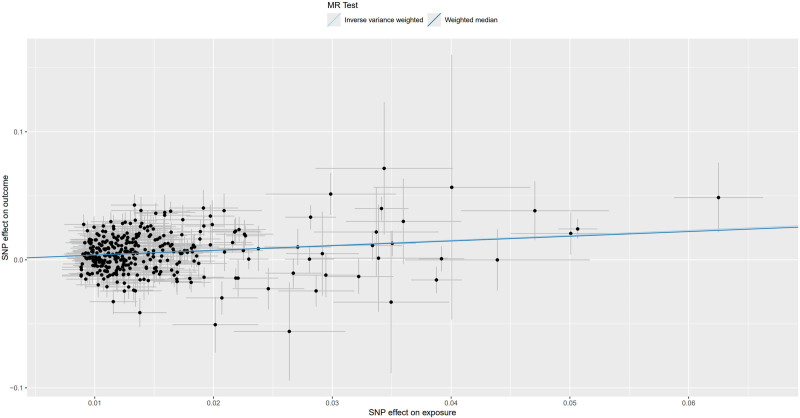
Scatter plot for MR analysis of right leg fat mass and IVDDs. IVDDs = intervertebral disc disorders, MR = Mendelian randomization, SNP = single nucleotide polymorphism.

**Figure 14.**
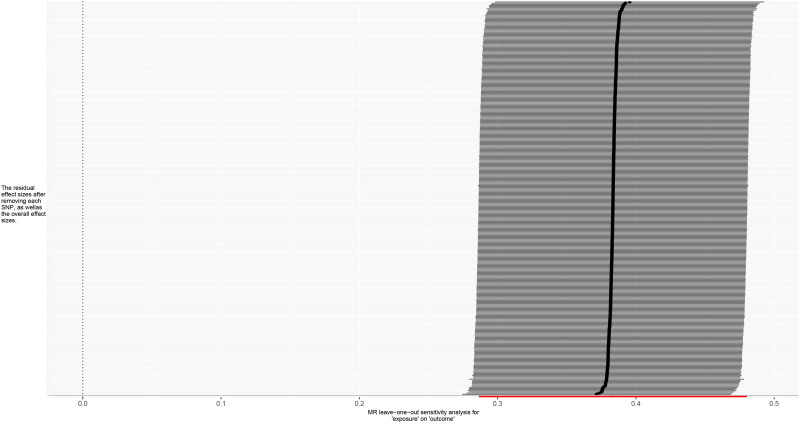
Leave-one-out forest plot for MR analysis of right leg fat mass and IVDDs. IVDDs = intervertebral disc disorders, MR = Mendelian randomization, SNP = single nucleotide polymorphism.

For right leg fat mass, 98,51,866 SNPs were read, and after using the clumping function to remove linkage disequilibrium, 421 SNPs strongly associated with right leg fat mass remained (*P* < 5 × 10^−8^). For IVDDs, 21,304,570 SNPs were read, and after merging with the SNPs strongly associated with right leg fat mass using the merge function, 413 SNPs remained. Using the harmonize function to eliminate duplicated, palindromic, and incompatible alleles SNPs, 339 SNPs remained. A review of the GWAS Catalog database found no interference from confounding factors, and the calculation of the *F*-statistic did not reveal any weak instrumental variables. Therefore, 339 SNPs were ultimately included as genetic variants for assessing the causal relationship between right leg fat mass and IVDDs. The Cochran’s *Q* test for the IVW method showed significant heterogeneity (*P* < .001), as did the Cochran’s *Q* test for the MR-Egger method (*P* < .001). Combined with the asymmetry observed in the funnel plot (Fig. 11), these results indicated the presence of heterogeneity in the study, necessitating the use of a random-effect model for statistical analysis. The research result from the IVW method was as follows: OR = 1.467, 95% CI: 1.332–1.616, *P* < .001, indicating statistical significance. Similarly, the result from the MR-Egger method was: OR = 1.456, 95% CI: 1.109–1.911, *P* = .007, showing statistical significance (Fig. 12 and Table S5, Supplemental Digital Content, https://links.lww.com/MD/P370). The direction of effect was consistent across both methods. With the IVW method’s result taking precedence, it could be concluded that an increase in right leg fat mass was a risk factor for the occurrence of IVDDs, indicating a positive causal relationship between left fat mass and IVDDs. The MR-Egger intercept test result (*P* = .953), along with the scatter plot (Fig. 13) showing a *y*-axis intercept close to 0, suggested the absence of potential in the study. A “leave-one-out” sensitivity analysis was conducted to examine whether the exclusion of individual SNPs would affect the overall causal inference. The analysis revealed that no individual SNP significantly impacted the prediction of the causal relationship (Fig. 14 and Table S6, Supplemental Digital Content, https://links.lww.com/MD/P370), indicating that the study’s result was relatively stable.

## 4. Discussion

This study, utilizing MR analysis based on data from the IEU open GWAS database and the FinnGen Biobank, explored the causal relationship between trunk and lower limb fat mass and IVDDs. The results indicated that an increase in both trunk fat mass and lower limb fat mass elevated the risk of IVDDs, identifying both as risk factors for the condition. This conclusion provided empirical support for the association between trunk and lower limb fat mass and IVDDs, offering insights for clinical prevention and treatment strategies.

Obesity is recognized as one of the pathogenic factors in spinal degenerative changes, primarily influencing IVDDs through adipokines released by fat and mechanical stress.^[30]^ BMI and fat mass are common metrics used to assess obesity, with localized fat mass proving more reliable for evaluating the degree of IVDDs in patients.

This study showed that increased trunk fat mass elevated the risk of IVDDs, primarily through 2 mechanisms: the release of adipose-derived substances and the mechanical stress exerted by additional body weight.^[31]^ The mechanism by which obesity induces osteoarticular diseases may involve lipotoxicity, defined as the accumulation of free fatty acids in non-adipose tissues.^[32,33]^ In pathological states, adipose tissue releases adipocytokines that not only participate in energy metabolism but also induce the liver to produce pro-inflammatory factors. These inflammation-related factors may lead to a change in the spinal inflammatory environment, causing localized pain similar to that observed in diabetic patients.^[34,35]^ Beyond the release of adipocytokines, the impact of trunk fat mass on IVDDs might also be linked to fat infiltration. The multifidus muscle, crucial for lumbar spine stability and resisting spinal movements like rotation, may have its function compromised when infiltrated by adipose tissue, leading to reduced control over external loads, such as shear and sliding forces, thereby failing to maintain structural stability of the disc and causing IVDDs.^[16]^

Similarly, the study noted that increased fat mass in the lower limbs also heightened the risk of IVDDs, affecting 2 main aspects: strength balance and support, and movement coordination. With the rise in fat mass, there could be a corresponding decrease in muscle mass or activity, as adipose tissue, while increasing body weight, does not contribute to muscular support. This reduction in muscle strength can place greater pressure on the lumbar spine, particularly affecting key muscles like the gluteus maximus and quadriceps, which are vital for pelvic stability and reducing lumbar stress. Additionally, an increase in fat can alter an individual’s gait and movement patterns, leading to uncoordinated actions that hinder the maintenance of the spine’s natural curvature and alleviate lumbar load, thereby increasing the risk of IVDD. Previous research on the link between lower limb fat and IVDD is scarce, but including it as a risk factor in the assessment of obesity’s impact on IVDD could propose new hypotheses and directions for research, offering better strategies for treatment and prevention.

Relying solely on observational studies to conclusively determine how fat mass and its distribution increase the risk of IVDDs and become a risk factor is impractical. This study employed MR as a novel epidemiological method, which offered several advantages: genetic variants used as instrumental variables were free from confounding factors such as social and lifestyle influences, making the results more reliable; these genetic variants, based on Mendel’s laws, preceded both exposure factors and outcome variables, thereby minimizing the possibility of reverse causation; MR data came from publicly accessible research literature and databases, allowing for large sample sizes without ethical issues and achieving a level of randomness comparable to RCTs, thus lowering research costs while maintaining credible results.

However, the study had its limitations. Primarily, the data from the IEU GWAS database and the FinnGen Biobank database represented European populations, which might limit the generalizability of the conclusions to other ethnic groups. The reliance on European population data in GWAS databases was a major limiting factor for the external validity of the results, as data from Asian populations and other regions were insufficient for large-scale analysis. The proliferation of DeoxyriboNucleic Acid sequencing and human genome technologies may help fill the gap in genetic variation data from other regions, potentially improving the prevention and treatment of human diseases. Moreover, the study did not differentiate between gender differences in abdominal and gluteal fat accumulation, which affected the shear forces the lumbar spine endures.^[36]^ Fat mass was only categorized into trunk and lower limb without specific distinctions between abdominal and lower back areas, limited by the lack of individual-level data in the IEU GWAS database. Strict selection of instrumental variables minimized sample selection bias, ensuring robust and reliable results. Finally, the validation part of the study indicated heterogeneity, yet a significant number of SNPs were maintained after rigorous filtering. An excess number of SNPs may be a major source of heterogeneity, but this did not detract from the interpretation of the study results. Future research would focus on further refining data sources and optimizing analytical tools to provide more reliable evidence for epidemiological studies.

In summary, this study using 2-sample MR revealed a positive causal association between trunk fat mass and IVDDs, as well as between lower limb fat mass and IVDDs. Previously, obesity assessments for disc degeneration primarily focused on BMI. This study’s genetic-level analysis around fat volume and its primary distribution offered a new perspective on how obesity impacts IVDDs, suggesting that future clinical interventions and prevention of IVDDs should pay closer attention to fat mass and distribution.

## Author contributions

**Conceptualization:** Zhihao Huang, Zhiqi Tian, Kunzong Tian.

**Formal analysis:** Zhihao Huang, Zhiqi Tian, Yuting Jiang.

**Investigation:** Zhiqi Tian.

**Methodology:** Zhihao Huang, Yongming Wang.

**Supervision:** Zhihao Huang, Zhiqi Tian.

**Visualization:** Zhiqi Tian, Kunzong Tian.

**Writing – original draft:** Zhihao Huang, Zhiqi Tian.

**Writing – review & editing:** Zhihao Huang, Zhiqi Tian, Kunzong Tian, Yongming Wang, Yuting Jiang.

## Supplementary Material


